# Local Application of Ibandronate/Gelatin Sponge Improves Osteotomy Healing in Rabbits

**DOI:** 10.1371/journal.pone.0125807

**Published:** 2015-05-07

**Authors:** Zongyou Yang, Wei Chen, Zhidao Xia, Yueju Liu, Shaun Peggrem, Tao Geng, Zhaoxu Yang, Han Li, Bin Xu, Chi Zhang, James T. Triffitt, Yingze Zhang

**Affiliations:** 1 Department of Orthopaedic Surgery, the Third Hospital of Hebei Medical University, Shijiazhuang, 050051 P R China; 2 Key Laboratory of Orthopaedic Biomechanics of Hebei Province, Shijiazhuang, 050051, P R China; 3 Orthopaedic Research Institution of Hebei Province, Hebei, P R China; 4 Centre for Nanohealth, College of Medicine, Swansea University, Singleton Park, Swansea, SA2 8PP, United Kingdom; 5 Department of Orthopaedic Surgery, Hebei General Hospital, Shijiazhuang, 050051, P.R. China; 6 Botnar Research Centre, Nuffield Orthopaedic Centre, Nuffield Department of Orthopaedics, Rheumatology and Musculoskeletal Sciences, University of Oxford, Oxford, United Kingdom OX37LD; Medical University of South Carolina, UNITED STATES

## Abstract

Delayed healing or non-union of skeletal fractures are common clinical complications. Ibandronate is a highly potent anti-catabolic reagent used for treatment of osteopenia and fracture prevention. We hypothesized that local application of ibandronate after fracture fixation may improve and sustain callus formation and therefore prevent delayed healing or non-union. This study tested the effect of local application of an ibandronate/gelatin sponge composite on osteotomy healing. A right-side distal-femoral osteotomy was created surgically, with fixation using a k-wire, in forty adult male rabbits. The animals were divided into four groups of ten animals and treated by: (i) intravenous injection of normal saline (Control); (ii) local implantation of absorbable gelatin sponge (GS); (iii) local implantation of absorbable GS containing ibandronate (IB+GS), and (iv) intravenous injection of ibandronate (IB i.v.). At two and four weeks the affected femora were harvested for X-ray photography, computed tomography (CT), biomechanical testing and histopathology. At both time-points the results showed that the calluses in both the ibandronate-treated groups, but especially in the IB+GS group, were significantly larger than in the control and GS groups. At four weeks the cross sectional area (CSA) and mechanical test results of ultimate load and energy in the IB+GS group were significantly higher than in other groups. Histological procedures showed a significant reduction in osteoclast numbers in the IB+GS and IB i.v. groups at day 14. The results indicate that local application of an ibandronate/gelatin sponge biomaterial improved early osteotomy healing after surgical fixation and suggest that such treatment may be a valuable local therapy to enhance fracture repair and potentially prevent delayed or non-union.

## Introduction

Fracture non-union is a common clinical complication which can affect 5–10% of surgically-treated fractures [1]. Non-union after fracture is difficult to heal without intervention. Current therapies for non-union comprise either non-surgical or surgical treatments. Surgical treatments for non-union involve the use of autografts, allografts or synthetic bone substitute biomaterials, with or without anabolic reagents such as bone morphogenetic proteins (BMPs) [2]. Patients who have suffered multiple fractures, extensive periosteal stripping, soft tissue damage, segment loss, infection or soft tissue necrosis are at high risk of non-union [3–7]. Currently, there is no available strategy which has the potential to prevent non-union in high-risk patients.

The normal course of fracture repair is a dynamic process involving a balance between modelling (intramembranous ossification and/or endochondral ossification to form callus), and remodelling (subsequent osteoclast-mediated resorption and osteoblast-mediated formation of callus) [8]. Imbalance of these processes is believed to be a key factor contributing to inadequate fracture healing. Reagents that inhibit bone resorption, such as bisphosphonates (BPs), and those that promote the formation of callus, such as BMPs, may be considered to offer potential beneficial effects on bone healing as callus provides a basic mechanical advantage to fracture stability. This is apparent in particular at the early stage of fracture healing [9–12]. Little (2007) has reported that the use of BP treatment alone is ineffective in the healing of non-union in a bone defect model; a combination of BP and BMP is needed [10]. However, the systemic or local application of BP in open osteotomy or in a closed fracture model significantly increased callus volume, callus bone mineral content, and mechanical strength of healing fractures when compared with non-treated rats [11, 12]. This implies that early stage application of BP can be beneficial for fracture healing, with potential preventive effects for delayed- or non-union.

BPs are synthetic analogues of pyrophosphate characterized by a P-C-P backbone with two variable side chains. They are highly potent inhibitors of osteoclast resorptive activity [8] and are major drugs used clinically for the treatment of postmenopausal osteoporosis [13], skeletal complications caused by malignant tumours [14, 15] and Paget’s disease [16]. BPs currently in clinical use can be separated into two categories, the non-nitrogen-containing BPs (etidronate and clodronate) and the nitrogen-containing BPs (risedronate, ibandronate, pamidronate, alendronate, and zoledronate). The modes of action of these two types on osteoclast activity are distinctly different, with the former being converted into non-hydrolysable ATP derivatives that can inhibit cellular enzymes dependent on ATP, and the latter inhibiting a key enzyme, farnesyl pyrophosphate synthase, resulting in disrupted cellular function [17].

BPs inhibit bone resorption by virtue of their selective adsorption to available bone mineral surfaces and subsequent being specific absorption by osteoclasts during bone resorption. This renders these cells inactive and eventually leads to their apoptosis with consequent preservation of bone density and volume [18, 19].

With oral administration of BPs various adverse effects have been reported, such as gastroduodenal ulcers, oesophageal events [20, 21], and, although rare, renal toxicity [22] and hypocalcaemia [23]. More adverse effects on the digestive [20], urinary [24, 25], hemic [26] cardiovascular [27] and musculoskeletal [28, 29] systems have been reported with the application of intravenous BPs. For example, the incidence of symptomatic hypocalcaemia is as high as 10% in patients given intravenous zoledronate, even when receiving prophylactic administration of vitamin D and calcium [26]. Acute phase responses (APR), characterized by symptoms of fever and myalgia, have been estimated to occur at a rate of 10% to 30% after the first infusion [30]. Animal studies have indicated that an intravenous dose of 3mg/kg of pamidronate promotes the mechanical strength of unification of healing fractures [9, 11, 31]. Pharmacokinetics showed that the bioavailability of bisphosphonates to inhibit bone resorption by intravenously delivery is limited to less than 1%- 2% absorption [32].

In previous studies on fracture healing, BPs have been applied systemically [31, 33]. There are some concerns that systemic administration may induce initial acute flu-like symptoms in some patients. In more vulnerable patients, for example those suffering from cancer and receiving very high doses of BP therapy, other possible side effects such as osteonecrosis of the jaw [34] or renal complications may occur [35]. Application of BP locally rather than systemically to a fracture may enhance the benefits of BP activity on skeletal preservation without the risks of potential systemic side effects. To ensure efficient delivery of BP to targeted skeletal tissue at the osteotomy site, without rapidly leaking to the surrounding tissues and the blood circulation, a carrier of BP for slow release is desirable. Gelatin sponge (GS) is a haemostatic material commonly used in orthopaedic surgery, and may be left at the application site, as it is bioresorbable. Its spongy nature makes it potentially suitable as a carrier for drug delivery. We hypothesized, therefore, that GS may be used as a local slow release carrier of BPs that would sustain callus formation following surgical treatment of fracture and thus benefit fracture healing. In the present study we have tested the hypothesis that ibandronate (IB), a widely used third-generation nitrogen-containing BP [36, 37] when combined with GS as a carrier and applied locally in a rabbit femoral osteotomy model improves initial skeletal healing.

## Materials and Methods

**Fig 1:**
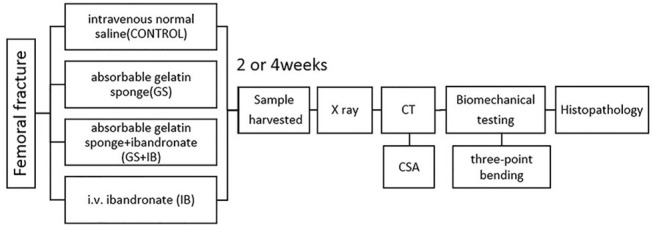
Brief flow chart of the experimental procedures.

The study was approved by the Regional Ethics Board of Hebei Medical University (Permit Number: L2013-001-1) and the institutional guidelines for the care and treatment of laboratory animals were rigorously followed. The experiments were conducted as outlined in Fig 1.

### Reagents and animal groups

Forty male New Zealand White rabbits weighing between 2 and 3 kg (aged between 12–16 weeks) were purchased from the Experimental Animal Centre of Hebei Medical University. The rabbits were randomly divided into 4 groups of 10 animals each. The groups were: (i) control group (intravenous injection of normal saline); (ii) GS group (local implantation of absorbable gelatin sponge); (iii) IB+GS group (local implantation of absorbable GS containing 0.25 mg ibandronate): (iv) IB group (0.1 mg/kg ibandronate, i.v.). To prepare IB+GS, 250 μL of 1 mg/mL sodium ibandronate in normal saline solution (Biomedical Engineering Centre of Hebei Medical University, China) was loaded onto one piece of absorbable gelatin sponge 60 mm × 20 mm × 5 mm (Jinling pharmaceutical Co., Ltd. Nanjing Jinling pharmaceutical factory, license number: H.M.L.N.H200110432) until the liquid was absorbed by the gelatin sponge, and subsequently lyophilized before use. For the GS group, 250 μL of normal saline (NS) was loaded onto absorbable gelatin sponge as described above and lyophilized. The dose of ibandronate used in the IB group, (group i.v.), was 0.1 mg per kilogram body weight and in the IB+GS (group iii), ibandronate was applied locally at 0.25 mg per rabbit. This dose is equivalent to the dose of 150 μg/kg of ibandronate reported by Manabe (2012) [38]. The animals were kept in individual cages under controlled environmental conditions (24 ± 2°C and 12-h light-dark cycle) with unlimited access to standard laboratory rabbit chow and water.

### Surgical procedures

The rabbits were anesthetized using sodium pentobarbital (3%, 1 mL/kg, Sigma-Aldrich, St Louis, MO, USA) and subsequently placed in the supine position. The right legs were selected as the side for operation, shaved and sterilized. A lateral incision along the distal femur was performed and the patella was dislocated medially to expose the femoral condyle. Through the inter-condylar approach, a sterilized Kirschner wire (ø 2.0 mm, Jinhuan Medical Products Co., Ltd, Shanghai, China) was drilled 7–8 cm into the medullary canal and the external part was cut off. Subsequently, a distal-femoral osteotomy was performed using dental drills (RA-II, 7.2V, Kangyu Medical instruments Co., Ltd, Guangzhou, China). The osteotomy sites in the GS group and the GS+IB group were surrounded with GS or IB-GS respectively and the patella was repositioned followed by suturing of the muscles and skin separately. The rabbits in the control group were injected with NS (0.1 mL/kg body weight) and the rabbits in the IB group were injected with ibandronate (0.1 mL/kg body weight in NS). After surgery, all animals received an intramuscular injection of buprenorphine (0.05 mg/kg) as analgesic twice a day and ciprofloxacin (10 mg/kg) antibiotic for three days. Body weight was measured every week and wounds checked for signs of infection daily.

### Evaluations

At 2 weeks after the operative procedure 5 rabbits were selected randomly from each group and were euthanized using a schedule 1 procedure, in which an overdose of barbiturates (100 mg/kg) was injected intraperitoneally. The remaining rabbits were euthanized at 4 weeks. The affected femora were harvested for the following examinations.

### X-ray Photography and CT

**Fig 2:**
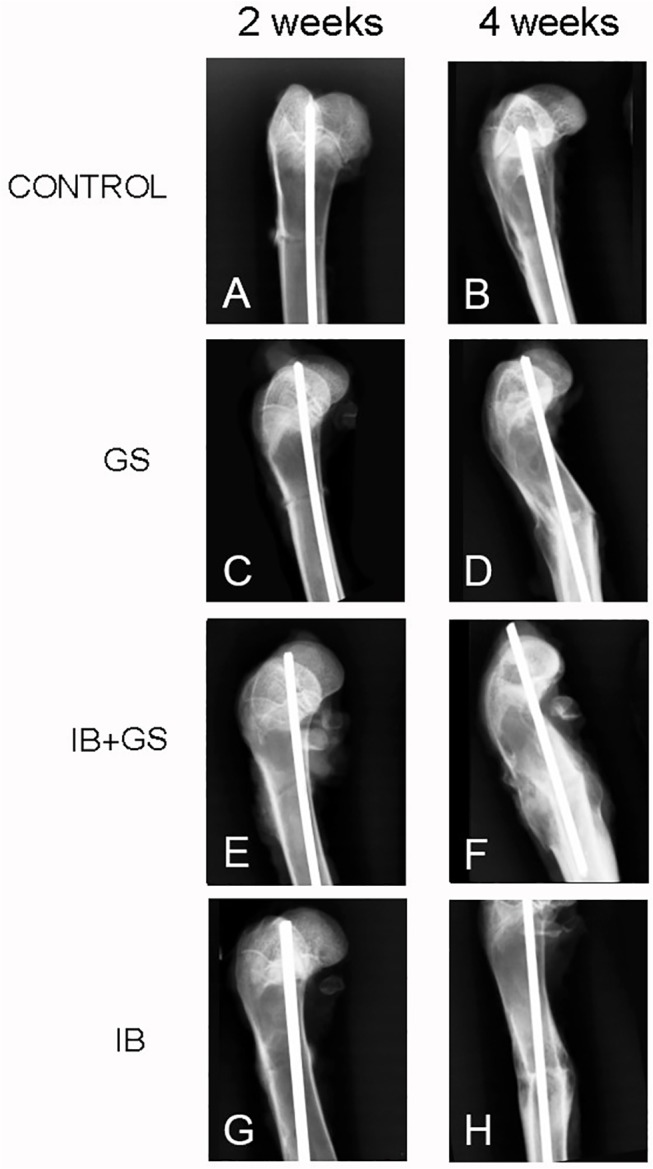
X-ray images at 2 and 4 weeks of the four experimental groups after fracture osteotomy.

**Fig 3:**
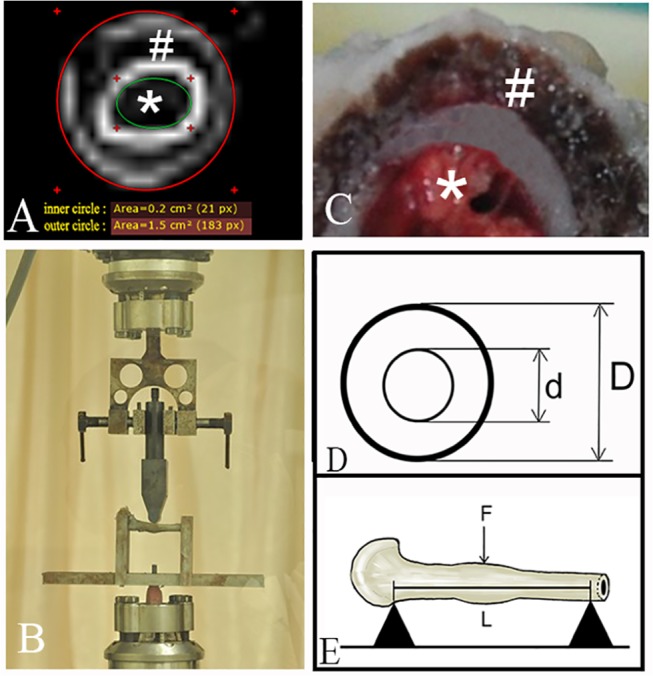
Illustration of CT-scan for CSA measurement, mechanical testing, and corresponding digital photograph of the fracture site. (A) A CT-scan image for CSA measurement. The outer circle (red) shows the outer surface of the callus, and the inner circle (green) shows the inner surface of the callus. CSA = outer circle area—inner circle area; (B) Illustration of mechanical testing by 3-point bending using the Bose (3520-AT) computerised system; (C) Typical photograph showing a single cross-section through the callus site formed after fracture from the mechanical test in (A). (D) The modelled cross section of the femoral samples. D, external diameter of samples; d, internal diameter. (E) Three-point loading. F, applied force; L, span length. # callus; * bone marrow.

The osteotomised femora were excised and the soft tissues dissected and discarded. Anteroposterior X-rays of the femora were imaged (20 kV, 10 mAs, Fujifilm, Tokyo, Japan) (Fig 2). The transverse and sagittal diameters of the callus at the osteotomy site were measured using CT-scanning (120 kV, 60mA, Siemens, Erlangen, Germany). The transverse plane from the CT scan nearest to the fracture line was selected to measure the cross sectional area (CSA) of the callus (Fig 3A). CSA was defined as total callus cross sectional area after deducting the area of the bone marrow cavity; therefore CSA is the cross sectional area for all callus, including external, internal and callus between osteotomy cortical bone fragments. The CSA in cm^2^ of the formed callus was measured using a RadiAnt DICOM Viewer 1.9 (Medixant, Poznan, Poland) (Fig 3A).

### Biomechanical tests

The femora were subjected to biomechanical testing by three-point bending (beam length 33 mm) using a computerized system (BOSE 3520-AT, Eden Prairie, MN, USA). Prior to this procedure the k-wire was removed (Fig 3B). The osteotomy plane of the femur tested was centred at the loading point and excessive proximal femur was removed for the convenience of loading in the apparatus. The force was applied in the sagittal plane at a rate of 10 mm/min until breakage occurred (Fig 3C). The mechanical data were evaluated to obtain the ultimate load (N), energy (Nmm) up to a 10% deviation from ultimate load along the deformation curve and stiffness (N/mm). The femur samples were modelled as thick-walled circular tubes (Fig 3D). The cross-sectional moment of inertia around the axis of bending, I, was calculated using the following equation:

$$I=\frac{\pi }{64}\left(D^{4}-{\text{d}}^{4}\right)$$ (1)

Where D is the external diameter of the sample and d is the internal diameter.

The equation for calculating Young’s modulus (MPa) is

$$E\;=\;\frac{\Delta F}{\Delta \text{d}}\frac{L^{3}}{48I}$$ (2)

where E is Young’s modulus, Δ*F*/Δd is the slope of the load-deformation curve, L is the span length (Fig 3E).

### Histopathology

After mechanical testing, the segments were carefully cut 0.5 cm from the osteotomy plane and fixed in 50 g/L paraformaldehyde (Zaolutang Pharmaceutical Group Co., Ltd, Xi’an, China) in PBS (pH 7.2–7.4) solution at 4°C for 3 days. The samples were then decalcified with 200 g/L EDTA solution at 4°C for 3–4 weeks, paraffin-embedded and sectioned (4 μm) using a microtome (Microm HM360, Waldorf, Germany). Ten non-overlapping fields of vision in the sections were routinely stained with hematoxylin-eosin (H&E, 0.5%) and used to evaluate the osteotomy healing process and the numbers of osteoclasts were subsequently counted in 5 fields of each sample on H&E stained sections at 2 and 4 weeks in each group.

### Statistical Analysis

All data are expressed as the mean ± SE. SPSS 13.0 statistical software (IBM, Armonk, NY, USA) was used for statistical analysis. One way analysis of variance (ANOVA) followed by Tukey's test was utilized to compare the differences of CSA, ultimate load, energy, Young’s modulus and osteoclast numbers among the groups, and was considered significant if *P* < 0.05.

## Results

No significant differences in body weight were found among the groups and animals resumed normal activity within two days after surgery. In the IB+GS group one rabbit was excluded because of death from diarrhoea.

### Radiologic findings

In X-ray images of the animals (Fig 2) at 2 and 4 weeks after osteotomy, fracture lines were still visible. However the fracture lines in both ibandronate-treated groups (especially in the IB+GS group) were less distinct and the callus larger than that observed in the other groups. At 2 and 4 weeks post operation, as seen in Table 1, the CSA, measured from the CT scan, in the IB group was significantly higher than that in the Control and GS groups; furthermore, the CSA in the IB+GS group was significantly higher than those seen in all other groups. There were no differences in these measurements between the Control group and the GS group (Table 1).

**Table 1:** Cross-sectional area (CSA, cm2) of callus at 2 and 4 weeks.

Group	2 weeks		4 weeks	
	n	CSA	n	CSA
Control	5	0.20±0.03	5	0.38±0.04
GS	5	0.18±0.04	5	0.40±0.03
IB+GS	5	0.62±0.04 ^a^ ^,^ ^b^	4	1.50±0.09 ^a^ ^,^ ^b^
IB	5	0.38±0.04 ^a^ ^,^ ^b^ ^,^ ^c^	5	0.84±0.07 ^a^ ^,^ ^b^ ^,^ ^c^
F	-	32.077	-	74.715
P	-	< 0.001	-	< 0.001

^a^ P < 0.05 vs. Control;

^b^ P < 0.05 vs. GS;

^c^ P < 0.05 vs. IB+GS.

### Biomechanical Tests

At 2 weeks after osteotomy the measurements of ultimate load, energy, Young’s modulus and stiffness did not significantly differ among all the groups (Table 2). At 4 weeks (Table 3) the ultimate load values in the IB group were significantly higher than those in the Control group or the GS group but were lower than in the IB+GS group. There was no difference between the Control group and the GS group. The parameters of energy in the IB+GS group were the highest, however, they were not significantly different when compared with the GS group. There were no differences in Young’s modulus among the four groups. In terms of stiffness, the IB+GS group exhibited significantly greater stiffness than the other three groups.

**Table 2:** Three-point bending (beam length 33 mm) of healing rabbit femoral fractures at 2 weeks.

Group	n	Ultimate load(N)	Energy(Nmm)	Young’s modulus (*10^2^ MPa)	Stiffness(N/mm)
Control	5	5.2±1.1	4.2±1.4	4.0±1.5	3.9±1.0
GS	5	6.1±1.0	5.9±1.5	6.7±1.9	4.8±0.8
IB+GS	5	9.0±2.0	7.8±2.0	8.6±2.6	7.1±2.4
IB	5	6.8±1.7	4.7±1.8	7.3±2.2	7.5±2.1
F	-	1.168	0.857	0.834	1.033
*P*	-	0.353	0.483	0.495	0.404

**Table 3:** Three-point bending (beam length 33 mm) of healing rabbit femoral fractures at 4 weeks.

Group	n	Ultimate load (N)	Energy (Nmm)	Young’s modulus (*10^2^ MPa)	Stiffness (N/mm)
Control	5	45.5±5.13	24.5±4.8	98.1±23.8	41.1±9.2
GS	5	49.9±6.7	33.6±7.1	88.2±19.5	45.2±6.0
IB+GS	4	129.8±9.6^a^ ^,^ ^b^ ^,^	88.4±9.2 ^a^ ^,^ ^b^	40.7±6.8	145.7±25.2 ^a^ ^,^ ^b^
IB	5	95.9±7.04^a^ ^,^ ^b^ ^,^ ^c^	64.6±7.3 ^a^ ^,^ ^b^	54.82±9.9	86.7±9.4 ^c^
F	-	30.738	16.199	2.358	13.067
*P*	-	0.000	0.000	0.113	<0.001

^a^ *P* < 0.05 vs. CONTROL;

^b^ *P* < 0.05 vs. GS;

^c^ *P* < 0.05 vs. IB+GS.

### Histopathology

**Fig 4:**
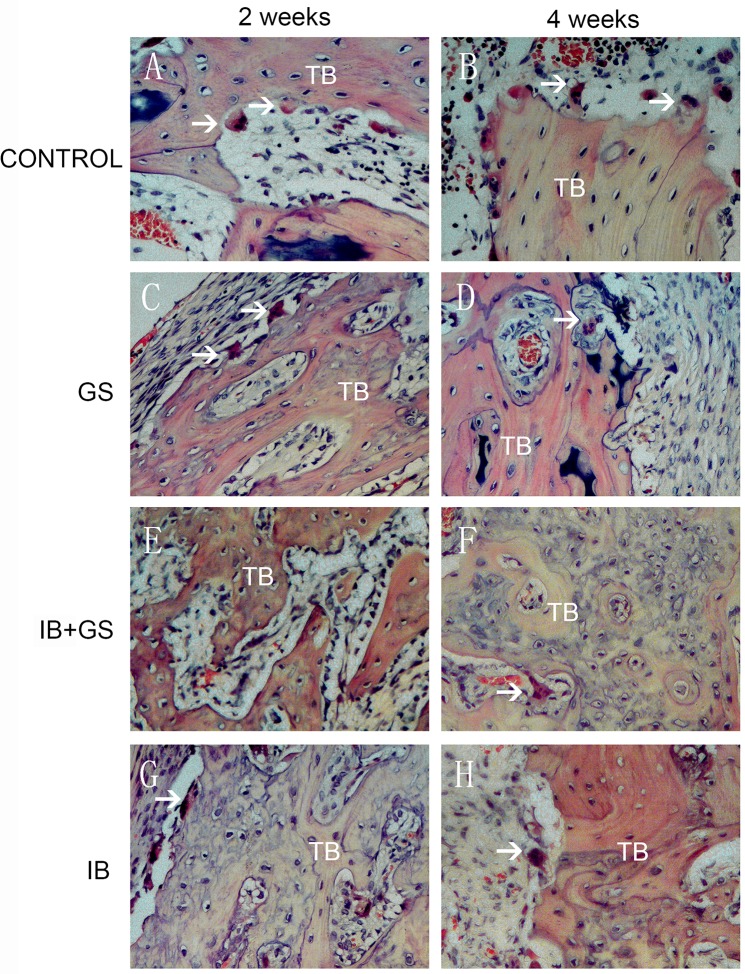
Photomicrographs of transverse sections through the fracture plane at 2 and 4 weeks after osteotomy, illustrating the presence of trabecular bone (TB) and osteoclasts (arrows).

**Fig 5:**
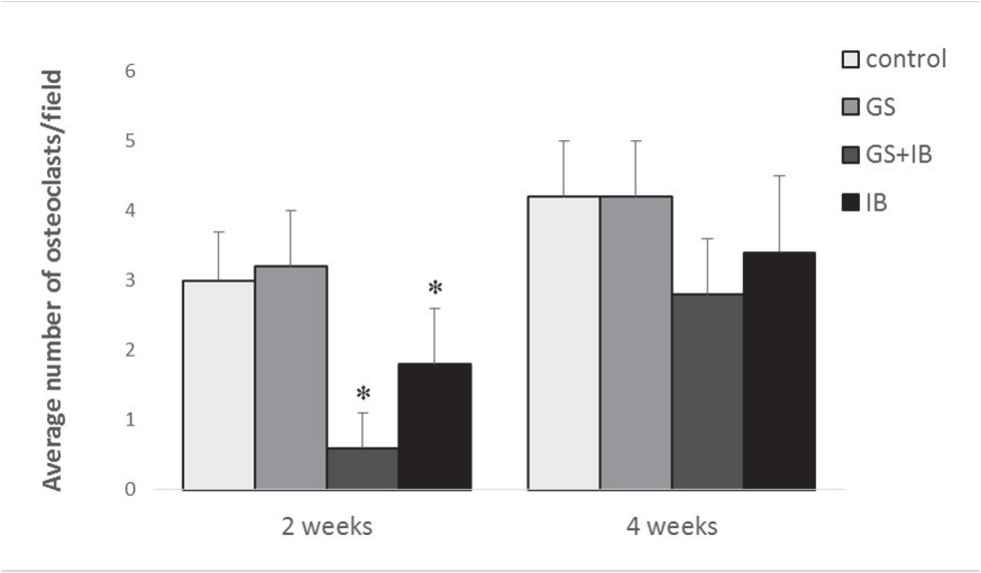
Osteoclasts measured on 5 non-overlapping fields of vision on H&E-stained sections at 2 and 4 weeks from the four groups. * Compared with the control, *P* < 0.05.for the GS group at 2 weeks.

At 2 weeks after osteotomy, histological sections of the osteotomy site in all groups showed that the site was occupied by calcified cartilage callus and fibrous callus. The callus in the IB+GS group was visually larger than that in the Control, GS or IB group (Fig 4, S1 Fig), which was consistent with the X-ray results. The number of osteoclasts seen in the callus of the IB+GS group was reduced significantly compared with the other three groups (Fig 5). At 4 weeks the callus in the IB+GS group was obviously larger and more tightly organised than in the other groups (Fig 4, S1 Fig). However, at this time-point osteoclasts were observed in all groups, with no differences in osteoclast numbers (Fig 5).

## Discussion

This study evaluated the efficacy of locally-applied ibandronate, combined with absorbable GS as a carrier, as a potentially beneficial treatment *in vivo* for enhancement of fracture healing. Locally-applied ibandronate is expected to bind readily to the available bone mineral at the fracture site. It is well known that BPs are anti-catabolics and have been reported to have no deleterious direct effects on the osteoblastic cells involved in bone formation. Bone formation at the site of fracture is a natural healing process. In a long bone fracture the callus is formed and organised via endochondral and intramembranous osteogenesis.

BPs have been found to be ineffective in promoting healing following non-union of critical bone defects in rats [9], but they can increase callus volume and improve mechanical properties when they are used at an early stage of open osteotomy and closed fracture [11, 12]. Early callus formation with increased mechanical strength is beneficial to fracture healing and helps avoid delayed healing and other healing complications [39]. Therefore, the purpose of the present investigation was to observe the effect of BPs on early callus formation in a rabbit osteotomy model when applied in combination with a local drug delivery carrier, rather than in the treatment of a non-union critical bone defect. In this study, we show that at 2 and 4 weeks after osteotomy, there are significant increases in external callus formation in animals treated with IB+GS when compared to the Control group, as determined by histological observation and measurements by X-ray photography. These findings are consistent with those reported in previous publications [11, 12, 38]. As IB is not expected to promote bone formation, we believe that this effect is due to inhibition of the bone resorbing cells, the osteoclasts, whose function is to resorb bone mineral during the course of fracture healing. In particular, at the callus remodelling stages they are known to act in the removal of mineralized cartilage callus formed in the process of endochondral ossification, and woven bone in intramembranous ossification. The processes of bone remodelling and fracture repair can be divided into anabolic (bone forming) and catabolic (bone resorbing) responses [40].

As the anabolic response results in bridging of the fracture by new bone, more attention has been paid to this process. However, catabolic action is also important as excessive or dysregulated catabolism may impede union. Osteoclasts are the cells responsible for bone resorption, are activated at an early stage of fracture healing and remain active until the end of remodelling and completion of healing [41]. However, in the case of aged patients or instances of fracture healing at sites where the environment is not ideal for bone formation, such as those with insufficient blood supply, excessive resorption and local inflammation may delay the union [3]. Anticatabolic agents such as BPs have previously been demonstrated to have beneficial effects in fracture healing by allowing increased callus formation [11]. This is achieved by inhibiting excessive callus resorption during the course of fracture healing. In this study, ibandronate was delivered locally and presumably binds avidly to the available bone mineral at the osteotomy site, due to the high affinity of ibandronate for bone hydroxyapatite. When this bone is resorbed by osteoclasts, the ibandronate is released from the bound state and internalized by the osteoclasts through the ruffled border [42]. Once internalized it interacts with its direct intracellular target, farnesyl diphosphate synthase, in the cholesterol biosynthetic pathway, resulting in disruption of a series of well-characterised biochemical processes important for osteoclast resorptive function.

Our results have shown quantitatively an increase in external callus formation. As revealed by radiographic evaluation, the volume of callus was larger in the locally applied ibandronate (IB+GS) group than in the other three groups, including the group administered intravenous ibandronate, at both time points studied.

Although callus size is important for bone healing, a key aspect of initial fracture stability and healing is the mechanical property of the callus. The ultimate aim of fracture healing is to restore bone strength, allowing it to bear mechanical loading. The mechanical properties of the healing osteotomies in both the ibandronate-treated groups were tested and showed significant improvements in ultimate loading at 4 weeks. This implies that both local and systemic ibandronate application increased the overall strength of the callus, enabling it to withstand mechanical loading.

There were no significant differences in Young’s modulus amongst the groups over 4 weeks, however, both energy and stiffness were significantly increased in the IB+GS group. This implies, from a mechanical property point of view, that the callus produced with local application of ibandronate was superior to that produced in the other groups, and is likely to promote accelerated fracture healing. Our results are consistent with this suggestion, and the beneficial effects are possibly due to two reasons. Firstly, the ibandronate in the local delivery group was delivered directly to the environment around the fracture fragments. Secondly, ibandronate in the intravenous administration group is likely to be present for a much shorter time and at a much lower concentration at the osteotomy site than in the locally-applied group. Since systemic delivery results in distribution of drugs throughout the body, there is likely to be a much more limited dose present in local tissue. Furthermore, intravenous ibandronate has a half-life (t_1/2_) of only 719 ± 200 minutes in the human with normal renal function [35].

There are concerns that although BP treatment is shown to be associated with shortening healing time to radiographic union for a week on average, the differences are too small to be considered clinically relevant [43]. Although there are visible reductions in osteoclast number by histological observation at 2 weeks after osteotomy, there are no differences in osteoclast number and morphology at 4 weeks after osteotomy. Whether this application has a long-term impact on callus remodelling, at a later time-point after osteotomy than observed here, should be investigated in future work.

### Limitations

In this work, biomechanical testing using the contralateral, non-fractured femur as an internal control was not performed. In addition, neither osteoclast-specific staining (TRAP staining) nor quantification of bone formation by histology, which could support the opinions more directly, were performed. In the animal model used, only male rabbits were selected which minimised hormonal effects (i.e. oestrogen) on the model. In addition in some rabbits the stabilizing pins migrated through the femoral condyles and changed the loading at the fracture sites. These limitations need to be considered for improvements in future work.

## Conclusions

The local application of a commonly used gelatin sponge as a carrier for ibandronate significantly increased osteotomy callus size and mechanical properties as determined by resistance to ultimate load and stiffness of the calluses after femoral osteotomy in the rabbit model. The findings indicate that local application of ibandronate/gelatin sponge can promote the early stages of fracture healing after surgical fixation. However, further research in non-union animal models and in the clinic are required before any future application for routine fracture therapy.

## Supporting Information

S1 Fig Photomicrographs of transverse sections through the fracture plane at 2 and 4 weeks after osteotomy, illustrating the presence of periosteum (PE), trabecular bone (TB) and medullary cavity (MC). (TIF)
