# Supplementary figures and images for: Local Application of Ibandronate/Gelatin Sponge Improves Osteotomy Healing in Rabbits
Source: PLoS One. 2015 May 7;10(5):e0125807. doi: 10.1371/journal.pone.0125807 (PMC4423918; doi:10.1371/journal.pone.0125807)

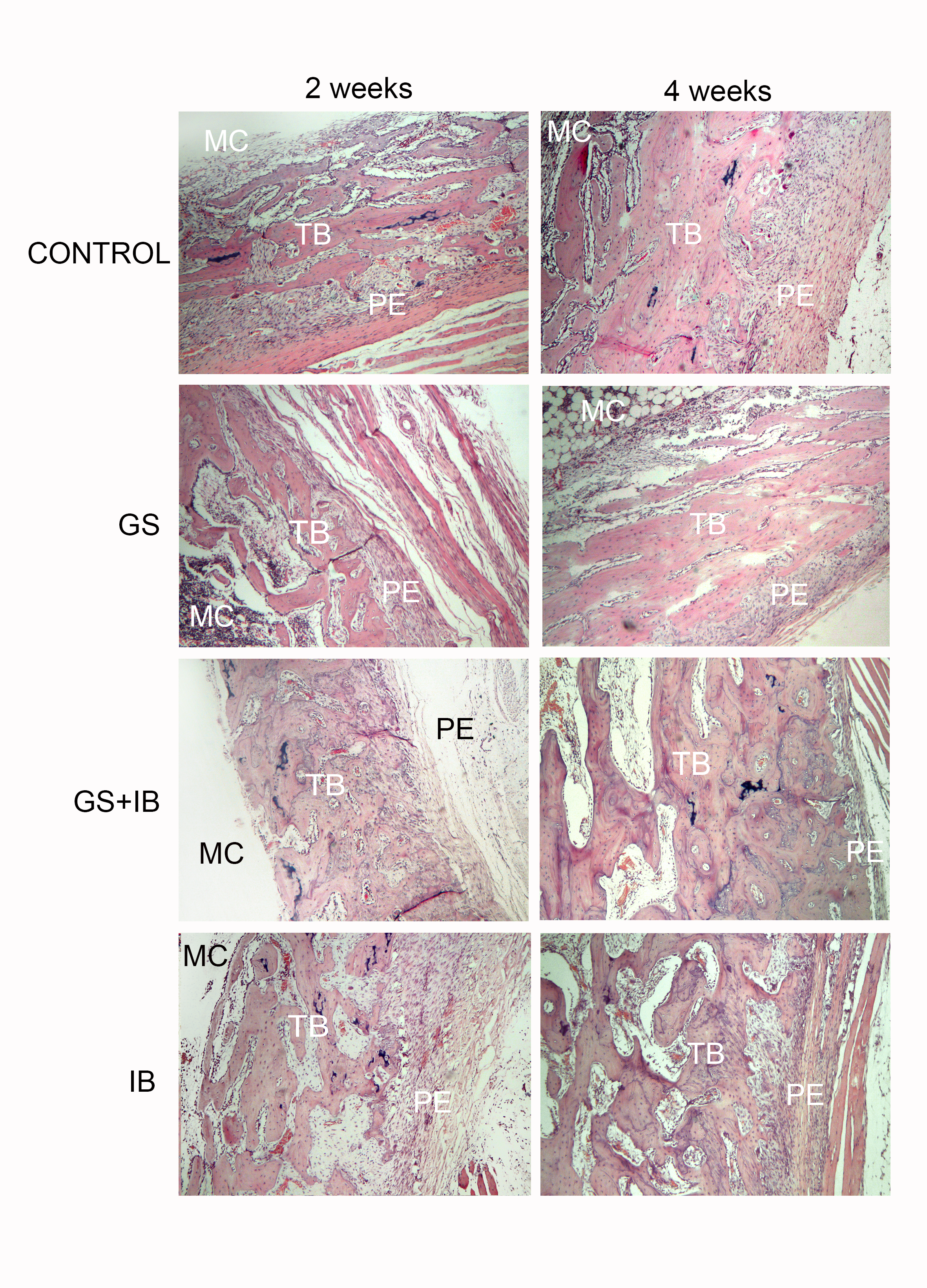

Supplement: S1 Fig — (TIF) [file pone.0125807.s001.tif]
